# Anaphylactic reaction as an etiology of ischemic stroke: A case report

**DOI:** 10.1016/j.radcr.2023.08.110

**Published:** 2023-09-26

**Authors:** Rakhmad Hidayat, Taufik Mesiano, Mohammad Kurniawan, Al Rasyid, Salim Harris, Ramdinal Aviesena Zairinal, Alyssa Putri Mustika, Gemia Clarisa Fathi, Aruni Cahya Irfannadhira

**Affiliations:** a Faculty of Medicine, Universitas Indonesia, Jakarta, Indonesia; b Dr. Cipto Mangunkusumo Hospital, Jakarta, Indonesia; c Universitas Indonesia Hospital, Jakarta, Indonesia

**Keywords:** Anaphylactic, Ischemic stroke, Anaphylaxis Reaction, Hypersensitivity

## Abstract

A 28-year-old man was brought to the emergency room (ER) with a history of decrease of consciousness 30 minutes before admission. In the previous 1 hour, the patient felt bitten or stabbed in the left temple area. Physical examination showed signs of N VII paresis, upper extremity motoric 3/2 (right/left) and lower extremity 3/2 (right/left), positive left extremity hypesthesia. Noncontrast MRI brain examination showed increased DWI signal intensity, suggesting diffusion restriction in bilateral centrum semiovale, bilateral posterior crus internal capsule, and bilateral corpus callosum leading to suspicion of acute-hyperacute ischemia. The therapy given while in the emergency room was IVFD asering, IV dexamethasone 5 mg, IV diphenhydramine 10 mg, oral paracetamol 500 mg, oral aspirin 80 mg, oral clopidogrel 75 mg, and oral atorvastatin 40 mg. We report a case of stroke due to an anaphylactic reaction in an effort to add to the point of view if the same case occurs.

## Introduction

Anaphylaxis is an acute reaction, involving various organs and very likely to be fatal [1]. The most common manifestations of anaphylactic reactions are in skin, respiratory, cardiovascular, and gastrointestinal. Anaphylactic skin reaction can reach 80%-90% of cases [2]. Manifestations of anaphylactic reactions are a combination of urticaria, erythema, pruritus, and angioedema [3]. There are several causes of anaphylaxis, including food, insect bites, and intravenous contrast fluids [2]. Insect bites more commonly only manifest in skin reactions rather than anaphylactic reactions. Anaphylactic reactions due to insect bites are estimated to reach 0.5%-3% per year [4].

Stroke is one of the highest causes of death in the world. Based on data from the American Heart Association/American Stroke Association (AHA/ASA) 2022, stroke occurs in 795,000 people/year, but those caused by insect bites is rare [5]. Various case studies have shown various clinical presentation, radiological results, complications, and outcomes. The outcomes in these cases varied from complete recovery to the vegetative state [6]. We report a case of stroke due to an anaphylactic reaction in an effort to add to the point of view if the same case occurs.

## Case report

A man, 28-year-old, was brought to the emergency room (ER) with a history of decrease of consciousness 30 minutes before admission. In the previous 1 hour, the patient felt bitten or stabbed in the left temple area. Complaints of itching appear in the area which then spreads throughout the body. The patient then looks weak, can talk but not coherent. He fell and hit his head, stiffened body, or seizure was denied. History of previous illness was denied.

**Fig. 1:**
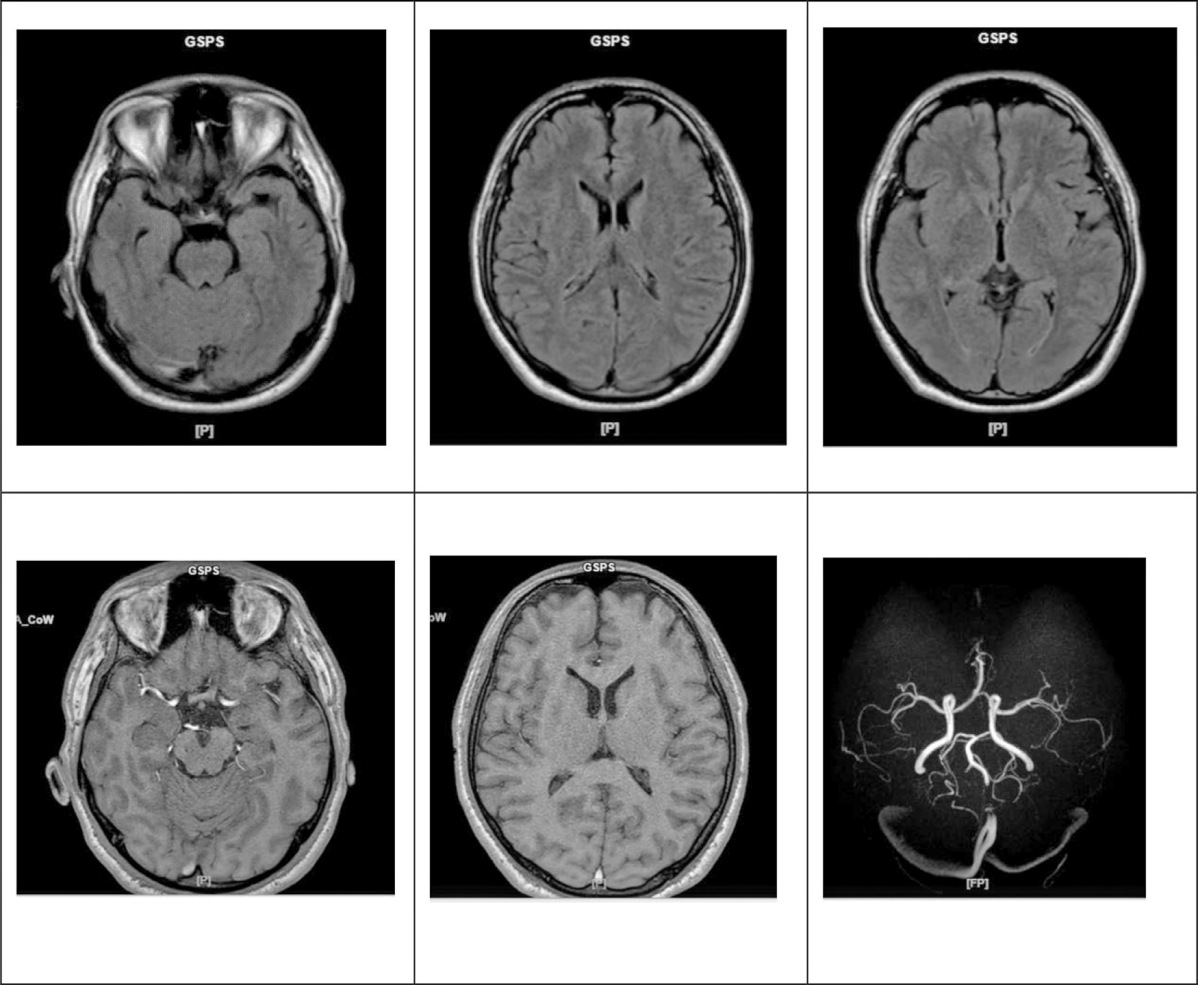
Result of noncontrast brain MRI.

When examined in the ER, the patient complained of headache and weakness on one side. Compos mentis consciousness, BP 124/65 mm Hg, HR 78 beats/minute, RR 20 times/minute, temperature 36.8°C, oxygen saturation 100% room air. Neurologic status GCS 15, NIHSS 7 (0/0/0/0/0/2/2/2/0/1/0/0/0), N VII paresis, upper extremity motoric 3/2 (right/left) and lower extremity 3/2 (right/left), positive left extremity hypesthesia, negative meninges stimulation, negative pathological reflexes. Hyperemic pharynx and erythematous rash on neck were found. On laboratory examination, the Hb value was 17.1, the hematocrit was 50.4 (Table 1). Platelet, leukocyte, electrolyte, and nonfasting blood glucose values were within normal limits. Chest X-ray examination showed no heart and lung abnormalities. Noncontrast MRI brain examination showed increased DWI signal intensity, suggesting diffusion restriction in bilateral centrum semiovale, bilateral posterior crus internal capsule, and bilateral corpus callosum leading to suspicion of acute-hyperacute ischemia (Fig. 1). The therapy given while in the emergency room was IVFD asering, IV dexamethasone 5 mg, IV diphenhydramine 10 mg, oral paracetamol 500 mg, oral aspirin 80 mg, oral clopidogrel 75 mg, and oral atorvastatin 40 mg (Table 2).

**Table 1:** Laboratory results.

Date		Value	Reference
June 22 2022 (The day of admission in the ER)	Complete blood count		

	Hemoglobin	17.1	13.0-17.0 g/dL
	Hematocrit	50.4	40.0%-50.0%
	Platelet	346,000	150,000-410,000/µL
	Leukocyte	5940	4000-10,000/µL
	MCV	83.9	83.0-101.0 fL
	MCH	28.5	27.0-32.0 pg
	Basophil	0.5	0%-2%
	Eosinophil	3.9	1%-6%
	Neutrophil	42.5	52.0%-76.0%
	Lymphocyte	48.7	20%-40%
	Monocyte	4.4	2.0%-10.0%
	**Electrolyte serum**		
	Sodium	137	132-147 mEq/L
	Potassium	3.85	3.3-5.4 mEq/L
	Chloride	103.7	94.0-111.0 mEq/L
	**Random blood glucose**	141	<140 mg/dL

**Table 2:** Neurological examination.

Neurological examination	ER (2 hours post onset)	Inpatient room (7 hours post onset)	(1 day post onset)	(2 days post onset)	(7 days post onset)	(14 days post onset)
Meningeal sign	None					
Consciousness/ GCS	CM/E4M6V5					
Pupil	anisochoric round pupil 4 mm/3mm	Isochoric round pupil 3 mm/3mm				
Cranial nerve	Paresis N.VII	Normal				
Physiological reflex	+2/+2 +2/+2					
Pathological reflex	None					
Sensoric	left hypesthesia	Normal				
Motoric	3333/2222 3333/2222	5555/3333 2222/2222	5555/4444 4444/4444	5555/5555 5555/4555	5555/5555 5555/5555	

On the second day of treatment, the weakness in the extremities improved. Complaints of numbness and headache were gone. Examination results showed normal cranial nerves, mild weakness on left motoric power.

On the third day of treatment, the weakness in the extremities had improved and they could be moved. The patient was then discharged. Medication given home included aspirin PO 1 × 80 mg, clopidogrel PO 1 × 75 mg, atorvastatin PO 1 × 40 mg, Cetirizine PO 1 × 1, Dexamethasone PO 2 × 0.5 mg, and Ranitidine PO 2 × 150 mg.

The patient was then come to the clinic to control after 7 days from the incident, complaints of weakness in the extremities of the body had improved a lot, began to be able to walk on the feet without dragging, grip strength was good. Complaint of numbness was denied. The patient's mRS was 1, the patient could carry out daily activities without significant obstacles even though there were still a few remaining symptoms.

On the 14th day after the first event, the patient came back to control. There were no complaints of weakness and the gait was back to normal as before the incident. Complaint of numbness was denied. The patient's mRS was 0, no body weakness.

## Discussion

Patient came with a history of decreased consciousness and weakness after being bitten by an insect 1 hour before admission. Examination revealed weakness in all 4 extremities, with the left side being weaker. There was left extremity hypesthesia. These various conditions lead to suspicion of stroke based on BE-FAST criteria (balance/leg weakness, eye/visual loss and diplopia, face, arm, speech, time) presumably due to anaphylactic shock [7].

After an insect sting, there is an increase in cytokines, especially interleukins such as IL-1, IL-6, IL-8, and tumor necrosis factor (TNF) which affect various locations in the body such as skeletal muscle, bone marrow, hepatic, renal, cardiovascular systems, central nervous system, and immune system. In this case, acute ischemic stroke can occur. Pathophysiologically, anaphylactic reactions involve the activation of mast cells which then produce chemical mediators such as histamine, lecotrin, triptat, and chymase. Histamine and leukotrienes are mediators that play a strong role in vasoconstriction of blood vessels, while triptat and chymase activate metalloproteinases which cause collagen degradation and plaque rupture [8].

Insect bites can cause various neurological manifestations such as stroke, epilepsy, poly-radiculopathy and cranial nerve lesions. The onset of these manifestations varied from 15 minutes to 4 days, with a median of 16 hours [6]. In this case, the manifestation of decreased consciousness occurred 30 minutes after the insect bite. The same onset was reported by Sancar et al. [9].

The result of noncontrast brain MRI showed suspicion of acute-hyperacute ischemia in the bilateral centrum semiovale, bilateral posterior crus internal capsule, and bilateral corpus callosum, supported clinically in the form of decreased consciousness and weakness in the extremities so that we concluded the patient had ischemic stroke due to anaphylactic shock. The study by Temizoz et al. [10] also showed MRI results in the form of lesion in bilateral centrum semiovale, in addition to ischemic in the frontal and temporoparietal lobes with clinical manifestations of left hemiplegia and dysarthria. Another study by Rehman et al. [11] with MRI result of lesion in the internal capsule's right temporal and posterior limb showing the same manifestation of left hemiplegia. MRI image in stroke due to allergic reactions was also reported by Sancar et al. [9] resembling ischemic stroke: hypertensive on DWI and hypotensive on ADC mapping.

Supporting laboratory results were Hb 17.1, hematocrit 50.4 indicating hypercoagulable state and erythrocytosis. Other results such as platelets, leukocytes, electrolytes, and nonfasting blood glucose were within normal limits. There are differences with several studies that show complete blood count (CBC) results within normal limits. Several studies have shown abnormalities in laboratory results in the form of increased urea, creatinine, LDL, triglycerides, SGOT, SGPT, and bilirubin (total, direct and indirect) [6,12]. Other abnormality which can be found in laboratory result in anaphylactic case is erythrosis or polycythemia which is generally relative. This relative polycythemia arises through several mechanisms: (1) vasodilatation of blood vessels causing extravasation of plasma to the extravascular; (2) hypoxia due to airway obstruction caused by airway edema or bronchoconstriction; (3) splenic contractions which increase circulating erythrocytes; (4) splanchnic congestion resulting in edema; or (5) loss of intravascular volume due to vomiting or diarrhea [13,14]. This condition is still very little reported in human studies and more reported in cases of insect bites that cause allergic reactions in animals.

The patient received therapy that focused primarily on anaphylactic reactions and acute ischemic stroke conditions, consisting of IV dexamethasone, IV diphenhydramine, cetirizine PO, and atorvastatin PO as initial therapy. The administration is based on the pathophysiology of anaphylactic reactions so that the main therapy is in the form of antihistamines (diphenhydramine and cetirizine) and anti-inflammatories (steroid) [15]. Although the main treatment for anaphylactic reactions is epinephrine and steroids, it is widely not recommended because there is no good quality evidence. The use of steroids has indicated a preventive effect on biphasic anaphylaxis [16]. In addition, atorvastatin was administered because of pleitropic effects including anti-inflammatory and plaque stabilization which can also to have developed[17].

The patient also received aspirin PO 80 mg, clopidogrel PO 75 mg, and atorvastatin PO 40 mg as secondary prevention. The study by Moein and Zand [6] also gave aspirin PO 325 mg and atorvastatin PO 80 mg in patient with manifestation of alien hand syndrome on the left hand with NIHSS 7 which then completely recovered in 4 days. Administration of aspirin PO 81 mg, and atorvastatin PO 40 mg were also given by Rehman et al. [11] to patient with clinical manifestations of left hemiplegia and NIHSS 2 who showed improvement within 1 month.

This patient did not receive thrombolytic therapy on the consideration that the ischemic stroke was based on an anaphylactic reaction. The patient came with manifestation of 1 hour onset of stroke, and NIHSS 7. This condition was seen as a criteria for activation of the stroke code system for the purpose of implementing thrombolysis but we did not do this even though NIHSS was >4. The patient's condition was caused by an anaphylactic reaction. Although anaphylactic conditions are not a contraindication to thrombolysis, the underlying pathophysiology includes activation of mast cells and chemical mediators (such as histamine, lecothrin, triptat, and chymase), as well as MRI features of diffusion restriction in bilateral centrum semiovale, bilateral posterior crus internal capsule, and bilateral corpus bilateral callosum so we decided not to do thrombolysis, but we gave dual antiplatelet therapy. This is different from what was done by Sancar et al. [9] who gave alteplase thrombolysis with doses starting at 0.9 mg/kg based on NIHSS values 7, but found no clinical improvement. Stroke due to anaphylactic reaction is similar to acute coronary syndrome due to anaphylactic (Kounsi syndrome), it caused by vasoconstriction due to the release of various inflammatory mediators so that giving thrombolysis does not solve the problem [18,19]. After treatment, the patient then experienced clinical and NIHSS score improvement.

## Conclusion

Stroke is a neurological emergency with a high mortality and morbidity rate. Anaphylactic reactions can end up in a state of shock which is also life threatening. Therapy for anaphylactic stroke is administration of antihistamines and anti-inflammatories. Stroke conditions due to anaphylactic reactions can have good outcomes by providing therapy based on clinical and radiological results of the patient.

## Patient consent

The authors have obtained informed consent from the relevant patient whose personal information and medical details are included in the manuscript.
